# Circulating Ageing Neutrophils as a Marker of Asymptomatic Polyvascular Atherosclerosis in Statin-Naïve Patients without Established Cardiovascular Disease

**DOI:** 10.3390/ijms231710195

**Published:** 2022-09-05

**Authors:** Vadim Genkel, Ilya Dolgushin, Irina Baturina, Albina Savochkina, Karina Nikushkina, Anna Minasova, Lubov Pykhova, Veronika Sumerkina, Alla Kuznetsova, Igor Shaposhnik

**Affiliations:** Federal State Budgetary Educational Institution of Higher Education “South-Ural State Medical University” of the Ministry of Healthcare of the Russian Federation, 454092 Chelyabinsk, Russia

**Keywords:** atherosclerosis, neutrophils, inflammation, aging neutrophils, CXCL12/CXCR4 axis

## Abstract

Background: Current data on the possible involvement of aging neutrophils in atherogenesis are limited. This study aimed to research the diagnostic value of aging neutrophils in their relation to subclinical atherosclerosis in statin-naïve patients without established atherosclerotic cardiovascular diseases (ASCVD). Methods: The study was carried out on 151 statin-naïve patients aged 40–64 years old without ASCVD. All patients underwent duplex scanning of the carotid arteries, lower limb arteries and abdominal aorta. Phenotyping and differentiation of neutrophil subpopulations were performed through flow cytometry (Navios 6/2, Beckman Coulter, USA). Results: The number of CD62L^lo^CXCR4^hi^-neutrophils is known to be significantly higher in patients with subclinical atherosclerosis compared with patients without atherosclerosis (*p* = 0.006). An increase in the number of CD62L^lo^CXCR4^hi^-neutrophils above cut-off values makes it possible to predict atherosclerosis in at least one vascular bed with sensitivity of 35.4–50.5% and specificity of 80.0–92.1%, in two vascular beds with sensitivity of 44.7–84.4% and specificity of 80.8–33.3%. Conclusion: In statin-naïve patients 40–64 years old without established ASCVD with subclinical atherosclerosis, there is an increase in circulating CD62L^lo^CXCR4^hi^-neutrophils. It was also concluded that the increase in the number of circulating CD62L^lo^CXCR4^hi^-neutrophils demonstrated moderate diagnostic efficiency (AUC 0.617–0.656) in relation to the detection of subclinical atherosclerosis, including polyvascular atherosclerosis.

## 1. Introduction

Currently, neutrophils, which account for the largest number among leukocyte populations, are considered to play an important role during the initiation, progression and complications of atherosclerosis [1,2]. A varying number of mechanisms have been identified and determined through which neutrophils realize their proatherogenic effects, these include: the production of reactive oxygen species (ROS), lipid mediators of inflammation, proteinases and cytokines; the formation of neutrophil extracellular traps (NETs); the orchestration of other cells responsible for innate and adaptive immunity as well as other mechanisms [3,4,5]. A large number of clinical studies have shown that both the absolute increase in the number of circulating neutrophils and the relative increase (as part of the neutrophil-to-lymphocyte ratio, NLR) are associated with the severity of atherosclerosis in different vascular beds and an increase in the relative risk (RR) of adverse cardiovascular events [6,7,8].

In the past few years, several discoveries in the biology of neutrophils have changed the traditional views on them as short-lived, terminally differentiated cells that predominantly perform the functions of phagocytosis at the initial stages of inflammatory response [9]. Neutrophils are a heterogeneous population of cells whose phenotypic plasticity determines various immunoregulatory functions that are realized in numerous biological compartments in health and disease [2,10,11,12]. All of these facts necessitate the further study of the role of specific subtypes of neutrophils in various chronic inflammatory diseases [2].

There are different approaches to the conceptualization of neutrophil heterogeneity, among which the identification of maturation status is widely used [13,14]. It is known that neutrophils change phenotype during their lifetime, which is commonly referred to as “neutrophil ageing” [15]. Neutrophil ageing has a significant impact on its functional features: the expression of CD62L decreases while the expressions of CD11b, TLR4 and CXCR4 increase as well as the ability to form NETs and produce ROS [16]. These features, which are present in this subtype of circulating neutrophils, may perhaps be an indicator of their potential involvement in the development and progression of atherosclerosis. First, the activation of the CXCL12/CXCR4 axis may serve to promote the recruitment and retention of circulating neutrophils in the subintimal space and atherosclerotic plaque [17,18]. Second, the increased ability of aging neutrophils to NET formation and ROS generation may help maintain chronic non-resolving inflammation in the atheroma, its growth and destabilization [19,20]. However, current data on the possible involvement of aging neutrophils in atherogenesis is limited, as is information regarding their potential diagnostic role in subclinical atherosclerosis. This study aimed to research the diagnostic value of aging neutrophils in relation to subclinical atherosclerosis, including polyvascular atherosclerosis, in statin-naïve patients without established atherosclerotic cardiovascular diseases (ASCVD).

## 2. Results

**Figure 1:**
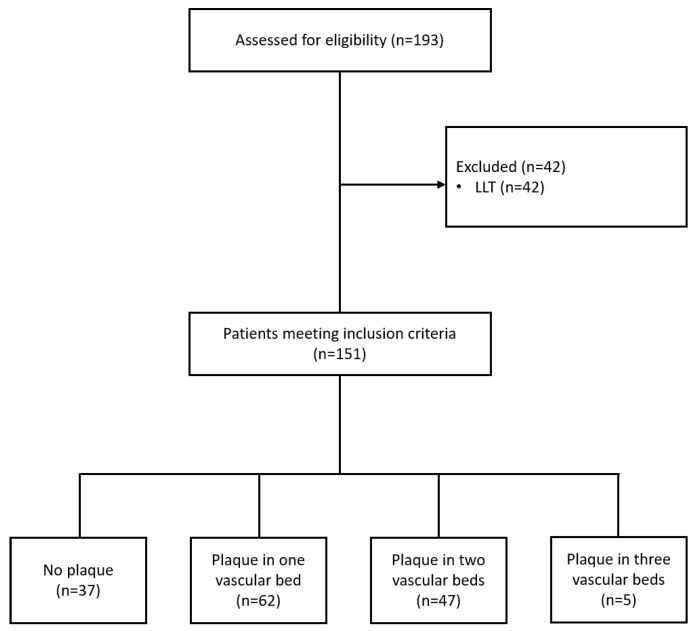
Study design.

The study was carried out on 151 patients who met the eligibility criteria. Of those, 64 were men and 87 were women (see Figure 1). 

As per the results of the duplex scanning of the carotid arteries, the lower limb arteries and the abdominal aorta, the patients were divided into four groups depending on the number of vascular beds in which atherosclerotic plaque was found.

The detailed clinical characteristics of the patients are presented in Table 1. 

As can be seen from the data presented in Table 1, patients with subclinical atherosclerosis were significantly older in comparison to patients without atherosclerotic plaque. Patients with polyvascular atherosclerosis (plaque in ≥2 vascular beds) had significantly higher total cholesterol and LDL-cholesterol compared with patients with intact arteries.

**Figure 2:**
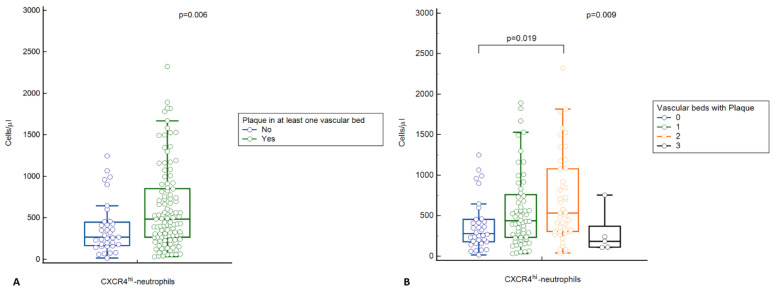
Number of circulating aging neutrophils according to the presence of atherosclerotic plaque in at least one vascular bed (**A**) or the number of affected vascular beds (**B**).

The results of flow cytometry are shown in Table 2 and Figure 2.

It should be noted that there was no significant difference in the total number of circulating neutrophils and mature neutrophils between the groups of patients in the study. In contrast, the number of CD62L^lo^CXCR4^hi^-neutrophils is known to be significantly higher in patients with subclinical atherosclerosis compared with patients without atherosclerosis (see Figure 2A). In addition, there was an increase in the number of CD62L^lo^CXCR4^hi^-neutrophils as the number of affected vascular beds increased from 0 to 2, followed by a decrease in patients with plaque in three vascular beds (see Figure 2B). 

**Figure 3:**
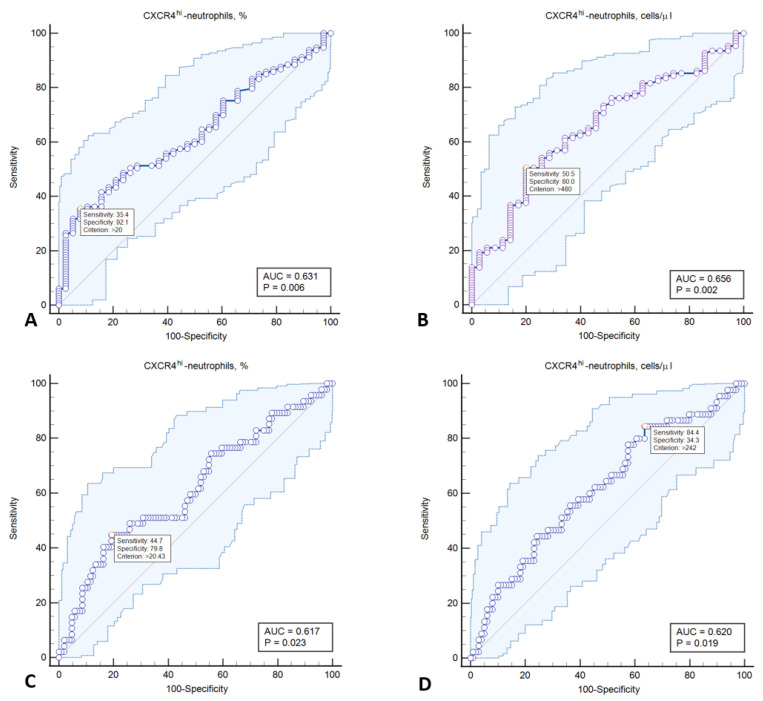
ROC curves demonstrating the diagnostic value of relative and absolute CD62L^lo^CXCR4^hi^-neutrophils counts in relation to the detection of atherosclerosis in one (**A**,**B**) and two vascular beds (**C**,**D**).

According to the results of the correlation analysis that was conducted, the absolute number of CD62L^lo^CXCR4^hi^-neutrophils directly correlated with maximal stenosis of carotid (r = 0.193; *p* = 0.021) and lower limb (r = 0.362; *p* < 0.0001) arteries. An ROC analysis was performed (see Table 3 and Figure 3) in order to evaluate the potential diagnostic value of CD62L^lo^CXCR4^hi^-neutrophils in relation to the diagnosis of subclinical atherosclerosis involving one and two vascular territories.

Given the data, it can be concluded that an increase in the number of CD62L^lo^CXCR4^hi^-neutrophils above certain threshold values makes it possible to predict, with a high degree of specificity, a subclinical atherosclerosis of at least one vascular bed in a patient without established ASCVD. Also worth noting are the high PPV values, which are at about 90%. Additionally, there is an increase in the relative CD62L^lo^CXCR4^hi^-neutrophils above 20.6% with high specificity, which makes it possible to predict the presence of polyvascular subclinical atherosclerosis. The highest values of the Youden index were obtained for the absolute number of CD62L^lo^CXCR4^hi^-neutrophils, which made it possible to predict polyvascular atherosclerosis with high sensitivity but low specificity, which, in turn, ensured high NPV values.

## 3. Discussion

Significant progress has been made in recent years in understanding the role of inflammation in the development of atherosclerosis. There is a growing body of evidence that inflammation is an integrating common pathway for the emergence of major downstream cardiovascular risk factors [21]. The widespread implementation of immunophenotyping of circulating cells into clinical research protocols and clinical practice is something that will continue in coming years and is aimed at solving the following main tasks: the identification of “inflammatory biosignatures” of the extent and the activity of subclinical atherosclerosis; the discovery of new targets for the anti-inflammatory therapy of atherosclerosis and the selection of patients with the highest likelihood of benefit from specific therapies; the discovery of new biomarkers of inflammatory residual risk in the framework of personalized medicine, as well as other tasks [21,22]. A separate direction in solving these problems is the study of the role of individual subtypes of circulating neutrophils in the development of atherosclerosis and other chronic inflammatory diseases [23,24].

The main results of the study being presented are as follows: (1) in patients aged 40 to 64 without established ASCVD and with subclinical atherosclerosis in at least one vascular bed, the number of circulating CD62L^lo^CXCR4^hi^-neutrophils was higher in comparison to patients without atherosclerosis; (2) as the number of affected vascular beds increased, there was a statistically significant increase in the number of circulating CD62L^lo^CXCR4^hi^-neutrophils; (3) an increase in the number of circulating CD62L^lo^CXCR4^hi^-neutrophils demonstrated a moderate diagnostic efficiency (AUC 0.617–0.656) in relation to the detection of subclinical atherosclerosis, including polyvascular atherosclerosis. In addition, the optimal cut-off value of the number of aging neutrophils to predict the presence of atherosclerotic plaques in at least one vascular bed was higher and consequently provided high specificity and PPV. In contrast, the optimal cut-off value to predict the presence of atherosclerotic plaques in two vascular beds was lower and provided high sensitivity and NPV. It should be noted that increasing the cut-off value in the range of 460–560 cells/μL in this setting failed to increase the specificity and PPV significantly. This is probably related to the substantial proportion of patients with a number of circulating aging neutrophils >460–560 cells/μL with plaques in only one vascular bed, which did not allow for achieving high specificity of the positive result. Therefore, the specificity of increasing the number of aging neutrophils was high in relation to predicting subclinical atherosclerosis but not its extent.

In our opinion, the results we obtained indicate the important role of the CXCL12/CXCR4/CXCR7 axis in the development of atherosclerosis. However, this is something that is currently not well understood [25,26]. While CXCL12 is considered to act primarily as a proatherogenic chemokine, activation of the CXCL12/CXCR4/CXCR7 axis can in fact lead to both the progression and stabilization of atherosclerosis [26,27,28]. This is probably largely determined by the fact that the CXCR4/CXCR7 expression is observed to be present on a large number of cells, such as neutrophils, monocytes, smooth muscle cells, endotheliocytes and others. The realization of the proatherogenic or athero-protective effects due to the activation of the CXCL12/CXCR4/CXCR7 axis will be directly dependent on the balance of CXCR4/CXCR7-expressing cells recruited into the vascular wall under the action of CXCL12 and further on the current stage of the atherosclerotic process [26]. For example, migration into the vascular wall of CXCR4/CXCR7-expressing smooth muscle cells acquiring a contractile phenotype can contribute to the stabilization of atheroma, while the recruitment of ageing CXCR4^hi^-neutrophils, on the contrary, can promote its growth [29]. It is possible that under the conditions of systemic persistent low-grade inflammation observed in atherosclerosis and an increase in the content of CXCL12 in the systemic circulation and atherosclerotic plaque, there is a disturbance in the migration of ageing neutrophils to bone marrow, an increase in their lifespan and blood content, followed by their recruitment into inflammatory foci in the vascular wall (which may also be associated with an increase in the expression level of adhesion molecules on ageing neutrophils) [17,30]. It has been established that large eccentric plaques and plaques with high inflammatory activity intensively accumulate CXCR4-expressing cells from the systemic circulation [31,32]. In addition, it is possible that as polyvascular atherosclerosis progresses, there is an increase in the systemic circulation of signaling molecules that trigger excessive and accelerated neutrophil aging, something that has been recently described as occurring in malignant tumors and HIV infections [33,34,35].

In a previous study conducted on a small mixed-sample of patients, we were able to establish the existence of relationships between an increase in the number of CD62L^lo^CXCR4^hi^-neutrophils and the extent of carotid atherosclerosis [36]. The present study has served to significantly expand on the previously obtained data with regard to the diagnostic value of aging neutrophils in relation to subclinical polyvascular atherosclerosis in statin-naïve patients aged 40 to 64. Although it must be noted that the presented study possesses a number of limitations: (1) due to an extremely small number of patients with atherosclerosis of three vascular beds, we were not able to judge the dynamics of the content of CD62L^lo^CXCR4^hi^-neutrophils with an increase in the number of affected vascular beds of more than two. Indeed, the involvement of the three vascular beds we studied is not very common in asymptomatic middle-aged patients. Given the 3-vessel rate (3.31%) observed in our group of patients, to include at least 20 patients would require a survey of about 600 patients; (2) there was no determination concluded as to the serum concentration of CXCL12.

According to the data we obtained, for the first time, an increase was demonstrated in the number of circulating CD62L^lo^CXCR4^hi^-neutrophils as the systemic burden of atherosclerosis increases, as well as the potential diagnostic value of aging neutrophils in relation to subclinical atherosclerosis. Indeed, further study is required of the diagnostic value of circulating CD62L^lo^CXCR4^hi^-neutrophils in relation to ASCVD in various groups of patients, as well as research into the role of aging neutrophils in the progression of atherosclerosis and the development of atherothrombotic events. Currently, active study of the targeting of the CXCL12/CXCR4/CXCR7 axis in the treatment of malignancy, rheumatoid arthritis, and multiple sclerosis is being carried out [37,38]. It is important to note that the use of the CXCL12/CXCR4/CXCR7 axis as a therapeutic target in the treatment of atherosclerosis has been shown to be complicated by the functional heterogeneity of CXCR4 on different cells and its involvement in the regulation of homeostasis, angiogenesis and tissue repair [26]. At the same time, there is evidence that has shown that the inhibition of interleukin-1 has led to a decrease in the relative risk of atherothrombotic events and in the development of new cases of lung cancer and can, at least partially, realize its effects through the suppression of CXCL12/CXCR4 signaling [39].

## 4. Materials and Methods

Statin-naïve patients aged 40–64 years of age without ASCVDs were enrolled in the study. A necessary condition for the inclusion of patients in the study was signed informed consent. The study protocol was approved by the Ethics Committee of the South Ural State Medical University (protocol No. 10, dated 27 October 2018). 

The following conditions were used as exclusion criteria for the study: previously diagnosed ASCVD (a history of cerebrovascular disease, coronary artery disease, peripheral arterial disease, revascularization of the coronary or peripheral arteries); severe liver and/or kidney dysfunction (marked by a decrease in an estimated glomerular filtration rate (eGFR) of less than 30 mL/min/1.73 m^2^); the presence of malignant neoplasms; established chronic inflammatory diseases; acute inflammatory or infectious diseases in the previous 28 days; the taking of statins and other lipid-lowering medications; the taking of any anti-inflammatory drugs.

### 4.1. Duplex Scanning

All patients underwent duplex scanning of the carotid arteries and the lower limb arteries. The study was carried out in B-mode, color mapping mode and pulse Doppler mode. The following vessels were examined from both sides in longitudinal and transverse sections throughout their length: the common carotid arteries (CCA) with CCA bifurcation, the internal carotid arteries (ICA), the external carotid arteries (ECA), common femoral arteries (CFA), superficial femoral arteries (SFA), the popliteal arteries (PA), the tibeoperoneal trunk, the anterior tibial arteries and the posterior tibial arteries. 

Atherosclerotic plaque was considered the focal thickening of the intima-media complex more than 1.5 mm or 0.5 mm larger than the surrounding intima-media thickness (IMT), or 50% more than the IMT of adjacent sections of the artery [40]. 

The percentage of stenosis was measured planimetrically in B-mode by diameter in the cross-section of the vessel. Stenosis percentages were determined according to the European Carotid Surgery Trial (ECST) method. In the case of the detection of plaque, stenosing the lumen of the vessels was carried out and the maximum percentage of stenosis in a particular patient was determined. The examination was carried out with a linear probe with a frequency of 10 MHz using the Canon Aplio 400 (Tokyo, Japan) digital ultrasonic multifunctional diagnostic scanner. 

An ultrasound examination of the abdominal aorta was carried out using a Canon Aplio 400 (USA) ultrasound scanner with a convex probe at a frequency of 3.5 MHz. The abdominal aorta was examined along its entire length, both in longitudinal and in transverse sections, from the proximal section below the diaphragm to the bifurcation. The study was carried out in B-mode, color mapping mode and pulse Doppler mode. Atherosclerotic plaque was considered as moderately or highly echogenic focal lesions encroaching into the lumen of the aorta [41,42].

### 4.2. Laboratory Tests

All patients underwent fasting blood count tests with an automatic analyzer (Medonic M16, Boule Medical A.B., Spånga, Sweden), for which their venous blood was collected into tubes containing K2 EDTA. 

The following biochemical laboratory blood parameters were obtained after fasting for at least 8 h: TC, LDL-C, HDL-C, TG, glycated hemoglobin, and creatinine with subsequent eGFR calculation according to the CKD-EPI formula. The concentration of high-sensitivity C-reactive protein (hsCRP) in blood serum was measured using an enzyme-linked immunosorbent assay (VECTOR-BEST, Novosibirsk, Russia). 

Phenotyping and differentiation of neutrophil subpopulations were performed through flow cytometry (Navios 6/2, Beckman Coulter, CA, USA). Blood was collected after fasting for at least 8 hours into K2 EDTA tubes. For the phenotyping of neutrophil subpopulations, conjugated monoclonal antibodies were used: CD16, PE-Cyanine7 (Invitrogen, Waltham, MA, USA); CD11b-FITC (Beckman Coulter, California, USA); CD62L-PE (Beckman Coulter, CA, USA); and CD184 (CXCR4)-PE-CF594 (BD Biosciences, NJ, USA). The gating strategy was described previously [43].

### 4.3. Statistical Analysis

The data that were obtained were analyzed using the statistical data analysis package MedCalc (ver. 20.019, MedCalc Software Ltd., Osten, Belgium) and IBM SPSS Statistics (v. 18, SPSS Inc., Chicago, IL, USA). Qualitative variables were described by absolute and relative frequencies (percentages). Quantitative variables were described by the median (Me), indicating the interquartile interval [25th percentile and 75th percentile]. Spearman’s correlation analysis was used to determine the relationship between the indicators. Any significant differences between more than two groups were assessed using the Kruskal–Wallis test followed by a pairwise comparison using the Mann–Whitney test. Cochran–Armitage’s Chi-square test for trend was used to assess the significance of differences in the frequency distribution of nominal variables between more than two groups. Differences were considered statistically significant if they were at a critical significance level of 0.05.

In order to establish the threshold values of the studied parameters, receiver operating characteristic (ROC) analysis was performed in order to obtain the determination of sensitivity, specificity, PPV and NPV. The calculation of the area under the characteristic curve (AUC) with a 95% confidence interval (CI) and Youden index was also carried out.

## 5. Conclusions

In statin-naïve patients aged 40–64 years old without established ASCVD with subclinical atherosclerosis, there is an increase in circulating CD62L^lo^CXCR4^hi^-neutrophils. It was also concluded that the increase in the number of circulating CD62L^lo^CXCR4^hi^-neutrophils demonstrated moderate diagnostic efficiency (AUC 0.617–0.656) in relation to the detection of subclinical atherosclerosis, including polyvascular atherosclerosis.

## Figures and Tables

**Table 1:** Clinical characteristics of patients.

Characteristics	No Plaques (*n* = 37)	Plaque in 1 Vascular Bed (*n* = 62)	Plaques in 2 Vascular Beds (*n* = 47)	Plaques in 3 Vascular Beds (*n* = 5)	Overall (*n* = 151)	*p*
Male, *n* (%)/Female, *n* (%)	15 (40.5)/22 (59.5)	23 (37.1)/39 (62.9)	24 (51.1)/23 (48.9)	2 (40.0)/3 (60.0)	64 (42.4)/87 (57.6)	0.416
Age, years, Me (LQ; UQ)	43.0 (40.0; 51.0)	48.0 (43.5; 53.0)	50.0 (46.0; 56.5)	56.0 (55.0; 56.0)	48.0 (43.0; 55.0)	<0.0001 *p*_1,0_ = 0.046 *p*_2,0_ < 0.0001 *p*_3,0_ = 0.001
BMI, kg/m^2^, Me (LQ; UQ)	25.3 (22.1; 28.7)	27.2 (23.4; 32.0)	25.9 (24.5; 29.2)	27.4 (25.7; 28.1)	26.0 (23.2; 29.0)	0.435
Obesity, *n* (%)	6 (16.2)	20 (32.2)	10 (21.3)	0 (0.00)	36 (23.8)	0.891
Abdominal obesity, *n* (%)	16 (43.2)	34 (54.8)	19 (40.4)	4 (80.0)	73 (48.3)	0.736
Smoking, *n* (%)	9 (24.3)	11 (17.7)	11 (23.4)	1 (20.0)	32 (21.2)	0.989
T2DM, *n* (%)	0 (0.00)	2 (3.22)	2 (4.25)	1 (20.0)	5 (3.31)	0.061
Hypertension, *n* (%)	11 (29.7)	24 (38.7)	20 (42.5)	3 (60.0)	58 (38.4)	0.135
Dyslipidemia, *n* (%)	30 (81.1)	53 (85.5)	44 (93.6)	5 (100.0)	132 (87.4)	0.051
Βeta-blockers, *n* (%)	3 (8.10)	5 (8.06)	5 (10.6)	1 (20.0)	14 (9.27)	0.428
Renin-angiotensin system inhibitors, *n* (%)	2 (5.40)	11 (17.7)	8 (17.0)	1 (20.0)	22 (14.6)	0.132
Diuretics, *n* (%)	0 (0.00)	4 (6.45)	3 (6.38)	0 (0.00)	7 (4.63)	0.308
Leukocytes, cells × 109/L, Me (LQ; UQ)	5.80 (4.78; 6.30)	6.20 (5.03; 7.18)	5.55 (4.65; 6.55)	6.03 (5.10; 9.05)	6.00 (4.85; 6.90)	0.483
TC, mmol/L, Me (LQ; UQ)	5.71 (4.94; 5.96)	6.02 (5.04; 6.90)	6.07 (5.37; 6.79)	6.28 (5.76; 7.50)	5.89 (5.14; 6.59)	0.026 *p*_3,0_ = 0.049
LDL-C, mmol/L, Me (LQ; UQ)	3.25 (2.90; 3.82)	3.64 (2.98; 4.62)	3.96 (3.28; 4.61)	4.03 (3.80; 4.23)	3.71 (3.02; 4.32)	0.009 *p*_2,0_ = 0.034 *p*_3,0_ = 0.038
HDL-C, mmol/L, Me (LQ; UQ)	1.41 (1.27; 1.61)	1.37 (1.17; 1.61)	1.29 (1.17; 1.63)	1.20 (1.19; 1.32)	1.37 (1.19; 1.61)	0.729
TG, mmol/L, Me (LQ; UQ)	1.07 (0.70; 1.40)	1.20 (0.81; 1.50)	1.35 (1.00; 2.10)	1.20 (0.80; 1.70)	1.20 (0.83; 1.60)	0.074
hsCRP, mg/L, Me (LQ; UQ)	2.48 (1.29; 3.00)	2.23 (0.73; 3.16)	2.56 (1.53; 3.05)	1.84 (1.61; 2.45)	2.50 (1.09; 3.11)	0.833
Glycated hemoglobin, %, Me (LQ; UQ)	5.48 (5.19; 5.74)	5.58 (5.17; 6.01)	5.79 (5.50; 6.24)	5.92 (5.40; 5.94)	5.61 (5.20; 6.00)	0.057
eGFR, mL/min/1.73 m^2^, Me (LQ; UQ)	75.0 (66.5; 84.0)	70.0 (65.5; 83.5)	73.0 (61.0; 100.5)	68.0 (68.0; 73.0)	72.0 (64.0; 87.0)	0.548
cIMTm, mm, Me (LQ; UQ)	0.61 (0.57; 0.66)	0.64 (0.55; 0.70)	0.67 (0.62; 0.74)	0.66 (0.66; 0.69)	0.65 (0.58; 0.70)	0.015 *p*_2,0_ = 0.015
Maximal carotid stenosis, %, Me (LQ; UQ)	0.00 (0.00; 0.00)	23.5 (20.0; 25.0)	25.0 (22.0; 31.0)	33.0 (33.0; 51.0)	22.0 (0.00; 28.0)	<0.0001 *p*_1,0_ < 0.0001 *p*_2,0_ < 0.0001 *p*_3,0_ < 0.0001 *p*_2,1_ = 0.033
Maximal stenosis of lower limb arteries, %, Me (LQ; UQ)	0.00 (0.00; 0.00)	0.00 (0.00; 21.0)	30.0 (25.0; 32.0)	30.0 (30.0; 35.0)	24.0 (0.00; 30.0)	<0.0001 *p*_2,0_ < 0.0001 *p*_3,0_ < 0.0001 *p*_2,1_ < 0.0001 *p*_3,1_ = 0.002

BMI = body mass index; TC = total cholesterol; HDL-C = high-density lipoprotein cholesterol; LDL-C = low-density lipoprotein cholesterol; TG = triglycerides; eGFR= estimated glomerular filtration rate; hsCRP = high-sensitivity C-reactive protein; T2DM = type 2 diabetes mellitus; cIMTm = mean carotid intima-media thickness; Me = median; LQ = lower quartile; UQ = upper quartile.

**Table 2:** Results of the circulating neutrophil count analysis.

Characteristics	No Plaques (*n* = 37)	Plaque in 1 Vascular Bed (*n* = 62)	Plaques in 2 Vascular Beds (*n* = 47)	Plaques in 3 Vascular Beds (*n* = 5)	Overall (*n* = 151)	*p*
Neutrophils, Absolute values, cells/μL, Me (LQ; UQ)	2.90 (2.41; 3.94)	3.50 (2.75; 4.25)	3.38 (2.63; 4.20)	3.60 (3.60; 4.80)	3.38 (2.60; 4.20)	0.540
Neutrophils, Relative values, %, Me (LQ; UQ)	57.0 (50.0; 66.0)	59.8 (48.1; 64.7)	57.8 (52.2; 63.7)	58.0 (50.0; 64.0)	58.0 (50.0; 64.2)	0.967
Mature neutrophils, Absolute values, cells/μL, Me (LQ; UQ)	2605.5 (1996.0; 3199.0)	3020.0 (2346.0; 3617.0)	2751.0 (2084.0; 3464.0)	2472.0 (2038.0; 5035.0)	2765.5 (2107.5; 3483.5)	0.478
Mature neutrophils, Relative values, %, Me (LQ; UQ)	87.0 (82.5; 92.1)	90.6 (82.2; 93.4)	88.4 (79.9; 93.9)	85.8 (74.2; 96.7)	89.2 (81.1; 93.4)	0.583
Ageing neutrophils, Absolute values, cells/μL, Me (LQ; UQ)	278.5 (178.0; 434.0)	438.0 (259.7; 791.0)	533.0 (300.0; 920.0)	184.0 (184.0; 539.0)	415.0 (231.0; 769.0)	0.009 *p*_2,0_ = 0.019
Ageing neutrophils, Relative values, %, Me (LQ; UQ)	12.3 (5.23; 15.9)	15.8 (6.66; 21.3)	17.6 (7.97; 27.6)	3.77 (3.15; 14.3)	14.3 (6.41; 21.0)	0.021 *p*_2,0_ = 0.038

Me = median; LQ = lower quartile; UQ = upper quartile; cells/µL = cells in 1 µL.

**Table 3:** Results of ROC analysis demonstrating the diagnostic value of aging neutrophils in relation to atherosclerosis in one and two vascular beds.

Characteristics	AUC (95% CI)	Cut-Off	Sensitivity	Specificity	Youden Index	PPV	NPV	*p*
Plaque in at least one vascular bed								
CD62L^lo^CXCR4^hi^-neutrophils, %	0.631 (0.548–0.708)	>20.0	35.4	92.1	0.275	93.0	31.8	0.0064
CD62L^lo^CXCR4^hi^-neutrophils, cells/µL	0.656 (0.572–0.733)	>460.0	50.5	80.0	0.305	88.7	34.1	0.0022
Plaque in two vascular beds								
CD62L^lo^CXCR4^hi^-neutrophils, %	0.617 (0.534–0.695)	>20.6	44.7	80.8	0.254	51.2	76.4	0.0228
CD62L^lo^CXCR4^hi^-neutrophils, cells/µL	0.620 (0.535–0.699)	>259	84.4	33.3	0.208	37.6	83.7	0.0193

AUC = area under ROC-curve; PPV = positive predictive value; NPV = negative predictive value.

## Data Availability

The data presented in this study are available on request from the corresponding author.
